# Pulse pressure and end-tidal carbon dioxide for monitoring low native cardiac output during veno-arterial ECLS: a prospective observational study

**DOI:** 10.1186/s13054-020-03280-z

**Published:** 2020-09-22

**Authors:** Marc Mourad, Jacob Eliet, Norddine Zeroual, Marine Saour, Pierre Sentenac, Federico Manna, Nicolas Molinari, Thomas Gandet, Pascal H. Colson, Philippe Gaudard

**Affiliations:** 1 Department of Anesthesiology and Critical Care Medicine, Arnaud de Villeneuve Hospital, CHU Montpellier, Univ Montpellier, Montpellier, France; 2 Epidemiology and Clinical Research Department, CHU Montpellier, Univ Montpellier, Montpellier, France; 3 Department of Cardiac Surgery, CHU Montpellier, Univ Montpellier, Montpellier, France; 4 Univ Montpellier, CNRS, INSERM, Institut de Génomique Fonctionnelle, Montpellier, France; 5 Univ Montpellier, CNRS, INSERM, PhyMedExp, Montpellier, France

**Keywords:** Cardiogenic shock, VA-ECLS support, EtCO_2_, Pulse pressure

## Abstract

**Background:**

Veno-arterial extracorporeal life support (VA-ECLS) results in cardiopulmonary shunting with reduced native cardiac output (NCO). Low NCO occurrence is common and associated with risk of thromboembolic and pulmonary complications. Practical tools for monitoring NCO during VA-ECLS would therefore be valuable. Pulse pressure (PP) and end-tidal carbon dioxide (EtCO_2_) are known to be related to cardiac output. We have designed a study to test whether PP and EtCO_2_ were efficient for the monitoring of NCO during VA-ECLS.

**Methods:**

In this prospective single-center observational study, patients who underwent a VA-ECLS for cardiogenic shock from January 2016 to October 2017 were included, provided low NCO was suspected by a PP < 20 mmHg. NCO was measured with pulmonary artery catheter or echocardiography and compared to PP and EtCO_2_. The ability of PP and EtCO_2_ to predict NCO < 1 L/min was evaluated with receiver operating characteristics (ROC) curves.

**Results:**

Among the 106 patients treated with VA-ECLS for cardiogenic shock during the study period, 26 were studied, allowing the collection of 196 study points. PP and EtCO_2_ relationships with NCO were nonlinear and showed strong correlations for NCO < 2 L/min (*r* = 0.69 and *r* = 0.78 respectively). A PP < 15 mmHg and EtCO_2_ < 14 mmHg had good predictive values for detecting NCO < 1 L/min (area under ROC curve 0.93 [95% CI 0.89–0.96] and 0.97 [95% CI 0.94–0.99] respectively, *p* = 0.058).

**Conclusions:**

PP and EtCO_2_ may offer an accurate real-time monitoring of low NCO events during VA-ECLS support. Further studies are needed to show if their utilization may help to implement therapeutic strategies in order to prevent thromboembolic and respiratory complications associated with VA-ECLS, and to improve patients’ prognosis.

**Trial registration:**

NCT03323268, July 12, 2016

## Introduction

Peripheral veno-arterial extracorporeal life support (VA-ECLS) for severe acute cardiac failure is increasingly used in patients with refractory cardiogenic shock (CS) or persistent cardiac arrest, either as a bridge to myocardial recovery, or to cardiac transplantation, or to long-term mechanical circulatory support [1–3]. However, although it provides good circulatory assistance, VA-ECLS has anti-physiological hemodynamic consequences, mainly, bypass of pulmonary circulation and backflow into the aorta. Indeed, VA-ECLS diverts venous return from the right atrium, reducing right ventricle pre-load, and returns blood into the aorta, increasing left ventricle (LV) afterload. It results in a proportional reduction of the pulmonary blood flow generated by the right and left ventricles (so-called, native cardiac output, NCO). Many adverse hemodynamic effects of VA-ECLS are related to these anti-physiological effects such as LV distension, pulmonary edema, and blood stasis in the pulmonary artery, cardiac chambers, or aortic root with high risk of thrombosis [4–6]. Moreover, low NCO during ECLS can induce significant pulmonary lesions and may be prevented by maintaining 25% of the systemic cardiac output through the pulmonary artery [7].

To overcome these drawbacks, current practices consist in tracking pulmonary edema, LV distension with spontaneous echo contrast (SEC), and aortic valve opening, mainly with repeated echocardiography exams [8]. Patients may then benefit from LV venting techniques to avoid the most serious complications [9–13]. The primary aim of venting techniques is LV discharge and prevention of pulmonary edema. Various techniques are proposed, with small differences in efficacy [14]. They differ in their ability to maintain or regulate NCO, but comparative data are not currently available. Therefore, monitoring NCO during VA-ECLS seems desirable to prevent the related complications. NCO is usually assessed by pulmonary artery catheter (PAC) with thermodilution measurement of right ventricle output, assuming the coupling of right and left ventricles results in the same output for both sides, and provided there is no left/right shunt. However, the technique is invasive and less reliable when cardiac output is low [15, 16]. Echocardiography is the alternative technique, either by directly measuring pulmonary artery flow (but this needs direct access to the pulmonary valve which is better viewed by trans-esophageal echocardiography) or by evaluating LV outflow by Doppler through the aortic valve (which can be obtained by transthoracic echocardiography). Nevertheless, whatever the echography technique, it cannot be considered as a monitoring technique. Often, the pulse pressure (PP) measured from the arterial line is used as a surrogate to assess NCO, but no study has yet addressed the issue of how much PP is sufficient. Similarly, end-tidal carbon dioxide (EtCO_2_) follows changes in cardiac output, provided CO_2_ production and removal are stable [17, 18]. In this respect, we recently observed a good correlation between EtCO_2_ and NCO in patients on VA-ECLS submitted to gradual increases of left-sided Impella assistance. The EtCO_2_ increase correlated quite well with the increased NCO assessed by echocardiography Doppler [19].

Based on these observations, we designed this study in order to assess the performance of PP and EtCO_2_ in evaluating NCO, and their accuracy to detect NCO below 1 L/min during VA-ECLS.

## Materials and methods

### Study design

This is a prospective, observational, cohort study, approved by our institutional review board (CPP Sud Mediterranée 1; ID RCB: 2015-A02006-43). Informed consent was obtained from all patients or their surrogates. The trial was retrospectively registered on Clinicaltrials.gov on July 12, 2016 (NCT03323268).

### Settings

The study was conducted in our tertiary hospital intensive care unit (ICU) from January 2016 to October 2017. Observations were recorded within the first 48 h after VA-ECLS implantation and up to 5 days after.

### Participants

All consecutive VA-ECLS patients admitted to our ICU during the study period were prescreened. Only the patients who experienced a PP < 20 mmHg were eligible, assuming the fact that PP < 20 mmHg could be considered, a priori, as a marker of low NCO. Patients were included if PAC was available. All included patients were followed afterwards over a maximum of 5 days, during which monitoring allowed several study points (see below) at various levels of NCO. Exclusion criteria included non-invasive ventilation at the time of screening, age less than 18 years, pulmonary disorders (obstructive pulmonary disease; acute respiratory distress syndrome, CS due to massive pulmonary embolism); intra-cardiac shunt (atrial or ventricular communication), and significant tricuspid or pulmonary valve disease. Left ventricular assist device (VAD) was not considered as exclusion criteria whenever VAD outflow was < 1.5 L/min.

VA-ECLS consisted of polyvinyl chloride tubing with a membrane oxygenator (PH.I.S.I.O and EOS; Sorin Group, Clamart, France), a centrifugal pump (Stockert; Sorin Group), and percutaneous or surgically inserted arterial and venous femoral cannulas (Fem-Flex and Fem-Track, Edwards Lifesciences, Guyancourt, France) with an additional 7F cannula inserted distally into the femoral artery to prevent lower limb ischemia. An oxygen-air blender (Sechrist Industries, Anaheim, CA) ventilated the membrane oxygenator. Unfractionated heparin was administrated to maintain an anti-factor-Xa activity of between 0.2 and 0.3 IU/mL.

In the initial phase of the circulatory assistance, VA-ECLS flow was set to provide adequate tissue perfusion (mixed venous oxygen saturation measured from the distal lumen of the PAC, SVO_2_ ≥ 65%) and to obtain correction of metabolic acidosis (serum lactate clearance). Thereafter, the VA-ECLS flow was set at the lowest rate necessary to ensure adequate tissue perfusion, while the highest NCO was wanted. Lung ventilation was managed with low levels of respiratory rate (10–14 breaths/min) and tidal volume (4–6 mL/kg), and with a modest level of positive end-expiratory pressure (8–10 cmH_2_O) to ensure protective ventilation [20]. Respiratory minute ventilation and ECLS sweep gas flow were adjusted to maintain baseline PaCO_2_ in a normal range, of around 40 mmHg.

In the case of severe LV distension, defined by pulmonary edema and/or threatening SEC on echocardiography, LV decompression (LV venting) was realized using transient left VAD (CP or 5.0 Impella devices, Abiomed Europe GmbH, Aachen, Germany).

### Outcome variables: native cardiac output, pulse pressure, and EtCO_2_

NCO was assessed by continuous (heated filament) thermodilution PAC (Swan-Ganz CCOmbo® CCO/SvO_2_, Edwards Lifesciences) inserted through the superior vena cava with placement confirmed by chest radiography. When NCO estimated by PAC was not possible (i.e., when the PAC monitor displayed “cardiac output < 1 L/min”), an evaluation of aortic and/or pulmonary outflow using Doppler echocardiography (transthoracic or trans-esophageal) was performed. An NCO threshold of 1 L/min was used to define threatening NCO (Th-NCO), considering NCO < 1 L/min at the higher risk of complications.

Continuous blood pressure was monitored via a radial arterial catheter. PP was defined as systolic arterial pressure-diastolic arterial pressure and studied only in the absence of concomitant left VAD.

EtCO_2_ was measured noninvasively from exhaled breath on a ventilator circuit and monitored using a ventilator CO_2_ analyzer (Maquet servo U, Drager Evita Infinity V500). Moreover, the arterial-to-end-tidal carbon dioxide (PaCO_2_-EtCO_2_) gradient was calculated as PaCO_2_ − EtCO_2_, PaCO_2_ being measured from arterial blood gas analysis (GEM4000premier®, Instrumentation Laboratory).

### Data collection

The following parameters were recorded: demographic characteristics, etiology of cardiac failure, context and patient severity at VA-ECLS implantation, hemodynamic variables and circulatory support at inclusion, and clinical course during VA-ECLS and follow-up.

Concomitant measurements of NCO, PP, and EtCO_2_ were realized in stable condition (no change in VA-ECLS and ventilator settings, patients’ treatment including hemodynamic supports and level of sedation). These study points were aimed first at ensuring that the VA-ECLS setting was optimal and also for catching Th-NCO events. The measurements were thus repeated, up to 4 a day, until NCO became > 2 L/min. Afterward, the measurements were made at the operator’s discretion, at least once a day, during the time of PAC monitoring.

The following data were also collected: hemodynamic support (catecholamine infusion, inhaled nitric oxide, cardiac pacing), left VAD outflow (if present), heart rate, systemic blood pressure, pulmonary artery pressures (PAP), pulmonary arterial wedge pressure (PAWP), SvO_2_, ventilator and VA-ECLS settings (respiratory mode, tidal volume, respiratory rate, positive end-expiratory pressure, plateau pressure, VA-ECLS, and sweep gas outputs), and blood gases sampled from the radial artery.

### Study size

We anticipated collecting data from eligible patients until obtaining at least 50 observations of Th-NCO.

### Statistical analysis

Categorical variables (expressed as absolute value and percentage) were compared using the chi-squared test. Continuous variables (expressed as median [25th–75th percentile]) were compared with Student’s *t* test or the Mann–Whitney *U* test, as appropriate according to the normality distribution assessed graphically.

The relationships of PP and EtCO_2_ with NCO were assessed graphically. The relationships of pulse pressure with stroke volume and PaCO_2_-EtCO_2_ gradient with NCO were studied to consider heart rate and PaCO_2_ confounders respectively. As these analyses showed nonlinear links for some variables, they were fitted in a regression model using a cubic spline [21]. The link between variables and the effect of uneven V/Q ratios (absolute difference to 1) at lungs and ECLS membrane on the relationship between EtCO_2_ and NCO were studied through an error prediction model (variations of model performance according to *X* or confounders). The NCO < 2 L/min level was identified as a cut-off value by calculating and comparing correlations from either side of several NCO levels using the weighted Spearman test and Zou’s confidence interval respectively.

The ability of PP and EtCO_2_ to predict Th-NCO was evaluated with receiver operating characteristics (ROC) curves and quantified by calculating the area under the curve (AUC) and 95% CI. A Delong test was used to compare matched ROC curves. The optimal threshold to predict Th-NCO was then determined as the one that minimized the explicit cost ratio, which is equivalent to maximizing Youden’s index. Statistical significance was defined as *p* < 0.05. The statistical analyses were performed using R environment (version 3.2.2, R Foundation, Vienna, Austria).

## Results

### Participants

**Fig. 1:**
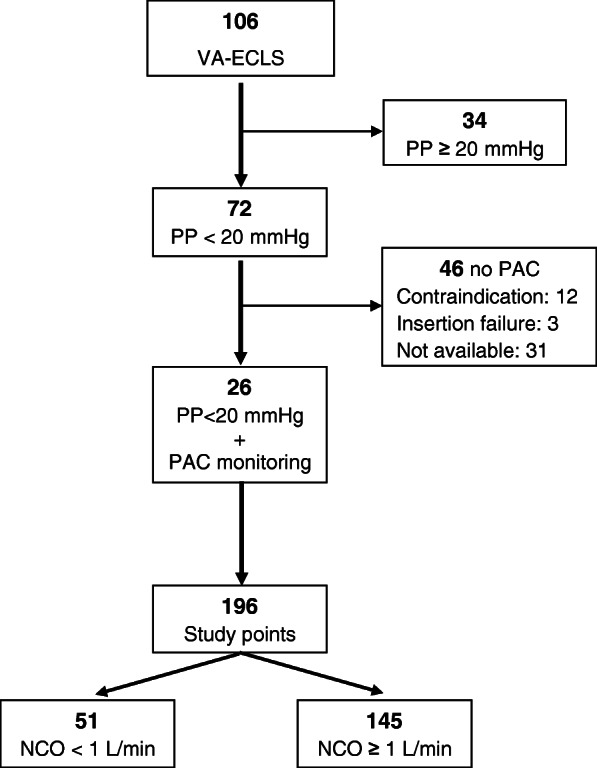
Flowchart. VA-ECLS, veno-arterial extracorporeal life support; PP, pulse pressure; PAC, pulmonary artery catheter; NCO, native cardiac output

Among the 106 patients treated with VA-ECLS during the study period, 72 presented an occurrence of PP < 20 mmHg and 26 had a PAC (Fig. 1, flowchart). Baseline patient characteristics, VA-ECLS management, and follow-up are displayed in Table 1. Five patients received VA-ECLS while already on transient or durable left VAD support and 4 patients required transient left VAD for severe LV distension during VA-ECLS.

**Table 1:** Patients’ characteristics, clinical course, and outcomes in intensive care unit

Men	16 (62)
Age, years	63 [58–70]
Body mass index	25 [23–29]
Cardiogenic shock etiology	
Post cardiotomy	11 (42)
Acute myocardial infarction	8 (31)
Dilated cardiomyopathy	5 (19)
0thers	2 (8)
Clinical and biological variables at VA-ECLS implantation	
Resuscitation before VA-ECLS	9 (35)
VA-ECLS under CPR	4 (15)
SOFA score	12 [12–13]
Durable left VAD	1 (4)
Transient left VAD	4 (15)
IABP	0 (0)
Serum lactate, mmol/L	7.1 [4.5–9.7]
Prothrombin time, %	56 [44–64]
Hemodynamic variables at inclusion (=1st study point)	
Heart rate, beats/min	90 [77–100]
Cardiac pacing	6 (23)
Norepinephrine, mg/h	3 [1.3–5.1]
Inotropic support	14 (54)
Inhaled nitric oxide	7 (27)
VA-ECLS flow, L/min	3.4 [2.9–4.1]
Left VAD flow if present, L/min	0.9 [0.4–1.5]
Clinical course in ICU	
Days under VA-ECLS	8 [6–10]
Add of transient left VAD during VA-ECLS	4 (15)
Add of IABP during VA-ECLS	0 (0)
Renal replacement therapy	10 (38)
Successful VA-ECLS weaning	18 (69)
Outcomes	
Length of ICU stay, days	21 [13–31]
ICU survival	13 (50)
6-month survival	11 (42)

Data are expressed as median [IQR interquartile range], or *N* (%)

*VA-ECLS* veno-arterial extracorporeal life support, *CPR* cardiopulmonary resuscitation, *SOFA* Sepsis-Related Organ Failure Assessment, *VAD* ventricular assist device, *ICU* intensive care unit, *IABP* intra-aortic balloon pump

### NCO measurements

Eight [5–9] measurements were realized per patient, totaling 196 study points. Fifty-one (26%) Th-NCO episodes were collected, mainly during the first hours (1 h [0–15]) of VA-ECLS. NCO ≥ 1 L/min study points (*n* = 145, 74%) were recorded later (median 30th hour [8–65]). Forty-seven (24%) of the study points were recorded while left VAD support was running (= missing data for pulse pressure), with a similar proportion between Th-NCO events and other study points (Table 2).

**Table 2:** Respiratory and hemodynamic data according to native cardiac output (NCO) < 1 L/min versus ≥ 1 L/min

	NCO < 1 L/min (*n* = 51)	NCO ≥ 1 L/min (*n* = 145)	*p* value
**VA-ECLS and ventilator data**			
VA-ECLS flow (Q ECLS), L/min	3.2 [2.9–4]	2.9 [2.2–3.8]	< 0.01
VA-ECLS sweep gas flow (V ECLS), L/min	5 [4–6]	4 [3–5]	< 0.01
V/Q ECLS	1.3 [1.2–1.7]	1.4 [1.1–1.8]	0.96
Tidal volume, mL	300 [260–350]	320 [300–360]	< 0.001
Respiratory rate, breaths/min	12 [11–14]	12 [11–14]	0.65
Ventilator minute volume (V lung), L/min	3.6 [3–4]	3.9 [3.5–4.7]	< 0.01
Positive end-expiratory pressure, cmH_2_O	10 [8–10]	10 [8–10]	0.91
Plateau pressure, cmH_2_O	18 [17–21]	18 [16–20]	0.26
V lung/V ECLS	0.8 [0.6–1]	1.1 [0.8–1.2]	< 0.001
**Hemodynamic data**			
Heart rate, beats/min	88 [73–107]	87 [72–106]	0.66
Right atrium pressure, mmHg	7 [5–8]	11 [8–13]	0.01
PA systolic pressure, mmHg	15 [12–18]	26 [23–34]	< 0.001
PA diastolic pressure, mmHg	12 [9–15]	19 [16–21]	< 0.001
Mean PA pressure, mmHg	13 [10–17]	21 [18–26]	< 0.001
PA wedge pressure, mmHg	10 [8–12]	14 [11–16]	0.01
NCO, L/min	0.5 [0.15–0.5]	1.8 [1.4–2.7]	< 0.001
Systolic BP, mmHg	79 [73–88]	95 [84–109]	< 0.001
Diastolic BP, mmHg	73 [67–79]	70 [63–77]	0.13
Pulse pressure, mmHg	9 [0–14]	31 [20–42]	< 0.001
Mean BP, mmHg	74 [69–80]	78 [72–87]	< 0.01
SvO_2_ (%)	74 [72–78]	72 [66–77]	0.10
**Respiratory data**			
PaCO_2_, mmHg	35 [33–40]	37 [34–41]	0.26
EtCO_2_, mmHg	9 [2–12]	23 [17–28]	< 0.001
PaCO_2_-EtCO_2_, mmHg	30 [22–33]	13 [8–19]	< 0.001
**Adjuvant treatments**			
Norepinephrine (mg/h)	3.2 [2.9–4]	1.2 [0.2–0.4]	< 0.01
Inotropic support	34 (67)	41 (28)	< 0.001
Inhaled NO	12 (23)	21 (14)	0.07
Transient or durable left VAD	11 (21)	38 (26)	0.57

Data are expressed as median [IQR interquartile range], or *N* (%)

*NCO* native cardiac output, *VA-ECLS* veno-arterial extracorporeal life support, *EtCO*_*2*_ end-tidal carbon dioxide, *PaCO*_*2*_*-EtCO*_*2*_ arterial-to-end-tidal carbon dioxide gradient, *BP* blood pressure, *PA* pulmonary artery, *SvO*_*2*_ mixed venous oxygen saturation, *NO* nitric oxide, *VAD* left ventricular assist device

Eighteen (69%) patients presented at least one episode of Th-NCO and 13 (50%) had Th-NCO at the first measurement. Respiratory and hemodynamic variables according to NCO < or ≥ 1 L/min are reported in Table 2.

### Pulse pressure and NCO

**Fig. 2:**
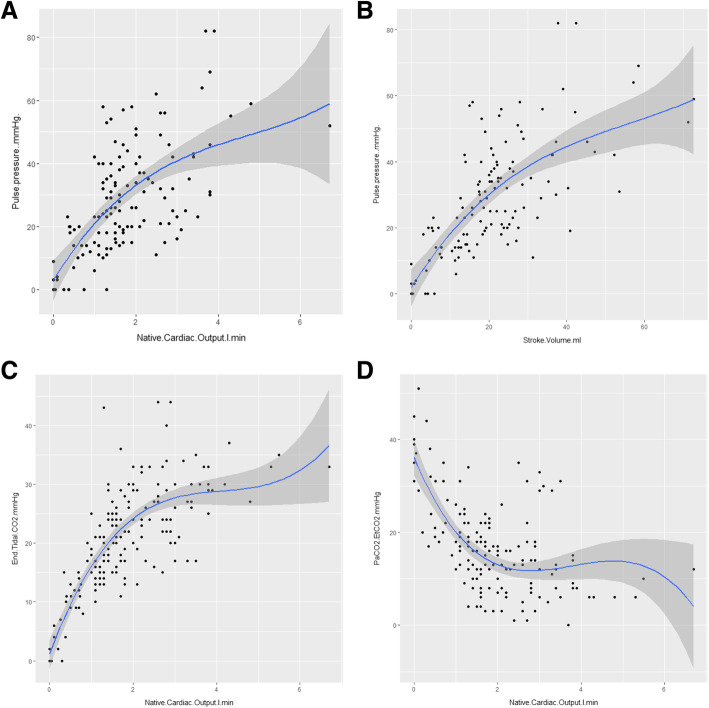
Pulse pressure and EtCO_2_ relationships with native cardiac output and their determinants. Native cardiac output was assessed with pulmonary artery catheter or echocardiography at the same time as pulse pressure, end-tidal carbon dioxide (EtCO_2_), arterial carbon dioxide pressure (PaCO_2_), and heart rate. Figures consist of spline regression representations (cubic spline, P Bruce and Bruce 2017) of the relationships between **a** pulse pressure and native cardiac output, **b** pulse pressure and stroke volume, **c** EtCO_2_ and native cardiac output, and **d** PaCO_2_-EtCO_2_ gradient and native cardiac output. The variation of model performance according to the *X* was evaluated through prediction of the error model (*p* < 0.001, p < 0.001, *p* = 0.01, and *p* = 0.3 for figures **a**, **b**, **c,** and **d** respectively)

As shown in Fig. 2a, PP relationship with NCO described a nonlinear regression curve. The spline regression model error increased significantly with NCO (*p* < 0.001 with positive coefficient), which means that the model fitted better for lower NCO values. A similar relationship was observed between PP and stroke volume, with the spline regression model error significantly increased with NCO (*p* < 0.001) (Fig. 2b).

Since the relationship between PP and NCO < 2 L/min was covering the NCO values of interest, we also tested a linear fitting, which showed a correlation coefficient of 0.694 [CI 0.570–0.786].

### End-tidal CO_2_ and NCO

As shown in Fig. 2c, EtCO_2_ relationship with NCO also described a nonlinear regression curve. The spline regression model error increased significantly with NCO (*p* = 0.01 with positive coefficient), which means that the model fitted better for lower NCO values. Indeed, linear fitting between EtCO_2_ and NCO < 2 L/min found a correlation coefficient of 0.779 [CI 0.683–0.848].

Of note, uneven V/Q ratios at lungs and membrane did not affect the relation between EtCO_2_ and NCO (*p* = 0.15 and *p* = 0.1 for V/Q ECLS and V/Q lung respectively).

A similar biphasic correlation was observed for the PaCO_2_-EtCO_2_ gradient but in the opposite direction to EtCO_2_ (Fig. 2d), and with weaker correlation coefficient (− 0.560 [− 0.685–0.402]) when considering the linear regression between PaCO_2_-EtCO_2_ gradient and NCO < 2 L/min.

### Accuracy of pulse pressure and EtCO_2_ to predict Th-NCO

The best cut-off values for predicting Th-NCO were 14.5 mmHg for PP (sensitivity = 0.83, specificity = 0.90, positive predictive value = 0.75, negative predictive value = 0.93) and 13.5 mmHg for EtCO_2_ (sensitivity = 0.88, specificity = 0.93, positive predictive value = 0.82, negative predictive value = 0.95).

**Fig. 3:**
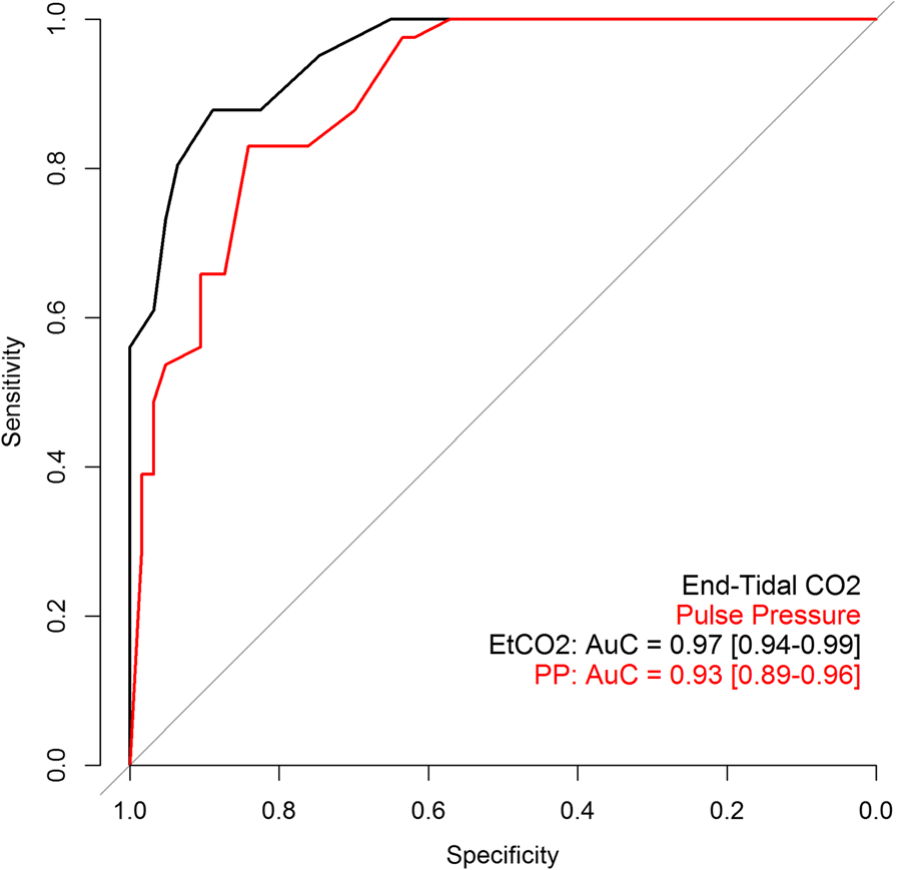
ROC AUCs of pulse pressure and EtCO_2_ for predicting native cardiac output < 1 L/min. ROC curve for pulse pressure (PP) in red and for end-tidal carbon dioxide (EtCO_2_) in black. Receiver operating characteristics (ROC) curves quantified by area under the curve (AUC) and 95% CI were obtained from 149 study points because 47 pulse pressure values were missing (patients on concomitant left VAD). *p* = 0.058 between ROC AUC of PP versus EtCO_2_ (Delong test)

ROC curve analysis of PP and EtCO_2_ cut-off values are shown in Fig. 3. ROC AUC tended to be higher for EtCO_2_ than for PP (0.97 [0.94–0.99] and 0.93 [0.89–0.96] respectively, *p* = 0.058).

## Discussion

This prospective study shows that, during VA-ECLS, PP and EtCO_2_ are strongly correlated with NCO when it is lower than 2 L/min. PP < 15 mmHg and EtCO_2_ < 14 mmHg predicted Th-NCO (NCO < 1 L/min) with good accuracy. Monitoring these parameters may help to prevent the risks associated with low NCO during VA-ECLS.

In the early phase of cardiovascular assistance in the case of severe CS, VA-ECLS is devoted to the restoration of blood flow to organs, a vital issue in avoiding multiple organ failure and death [22]. Therefore, low residual transpulmonary blood flow or NCO is common during the initial hours after VA-ECLS implantation [23]. In our series, owing to the fact that patients were included with PP < 20 mmHg, a high incidence of Th-NCO was expected and 13 (50%) patients did indeed have Th-NCO at the first measurement. However, most Th-NCO episodes (*n* = 38, 75%) occurred afterwards, despite global NCO improvement over time.

Not surprisingly, Th-NCO was associated with higher VA-ECLS flow, lower blood and pulmonary artery pressures, stable heart rate, and higher inotropic and vasopressor support (Table 2), a hemodynamic profile that underlines a high dependence on VA-ECLS. However, we also observed lower right atrial pressure and lower PAWP when NCO was < 1 L/min compared to NCO ≥ 1 L/min, suggesting that hypovolemia may have worsened the NCO. High VA-ECLS outflows are at greater risk of pulmonary circulation exclusion and left ventricle distension, and thus, related complications may occur. The study was not designed to explore these complications, but several publications have already reported risk of blood stasis, stroke incidence, or intra-cardiac or aortic root thrombosis [5, 6, 24, 25]. In our series, 4 (15%) patients needed LV venting with Impella to avoid major complications, an incidence in agreement with previous studies [12, 13]. Poor prognosis of Th-NCO increases with its duration or episode repetition [7, 23]. Therefore, the monitoring of NCO is strongly recommended to avoid a prolonged cumulative time of Th-NCO [8].

In this respect, ELSO guidelines recommend the use of a PAC to maintain mean PAP > 30 mmHg, echocardiography to avoid non-opening of the aortic valve, and continuous arterial monitoring to track the non-pulsatile arterial line [8]. PAC is mandatory for measuring PAP, but it is an invasive monitoring method with a limited duration of utilization due to septic risk [26]. High PAP may also reflect left ventricular overload, through an elevation of PAWP, a major concern during VA-ECLS. Moreover, in our series, even when NCO exceeded 1 L/min, mean PAP was lower than 30 mmHg, which indeed seems like a rather difficult objective to achieve when patients are very VA-ECLS dependent. Incidentally, PAC with continuous thermodilution measurement of cardiac output cannot measure flows < 1 L/min and becomes less reliable when cardiac output falls below 2 L/min, which makes PAC less useful in the condition of high level of ECLS assistance [15, 16]. The PAC limitation on cardiac output measurement in low flow states could be overcome with adaptation of the thermodilution technique, mainly with resetting catheter constant at various flow ranges [27]. Although attractive, the technique is currently not available in clinical practice.

A continuous monitoring of the arterial pressure line can evidence loss of pulsatility, and echocardiography may then confirm absence or rare openings of the aortic valve. The loss of pulsatility is associated with blood stasis and a high incidence of stroke (41%) [6]. However, analysis of PP may go beyond the qualitative evaluation of the presence or absence of pulsatility. In physiology, PP is linearly related to the stroke volume and inversely related to arterial compliance [28]. In the non-physiological condition of the VA-ECLS, PP was still strongly related to stroke volume, especially for lower stroke volume values. The NCO relationship with pulse pressure followed that of stroke volume, in agreement with a heart rate that remained constant. The weaker relationship with upper values might be explained by a reduction of VA-ECLS backflow pressure. VA-ECLS outflow was reduced proportionally to the improvement of NCO, therefore decreasing LV afterload, which is equivalent to an increase in arterial compliance [28]. Anyway, the good relation between low NCO levels and PP was exemplified by the ROC curve analysis, with excellent accuracy for predicting Th-NCO. However, PP monitoring may be limited by any venting technique involving the left ventricle, by aortic valve leaks, or by the presence of an intra-aortic balloon.

Conversely, EtCO_2_ appears as a very pertinent parameter, even when PP is not reliable. EtCO_2_ monitoring is a routine and non-invasive measure in ventilated patients. Recent studies have found a correlation between NCO and EtCO_2_ in patients requiring LV venting during VA-ECLS [19, 29]. In the present study, we demonstrate that EtCO_2_ correlated strongly with NCO when < 2 L/min, and even that EtCO_2_ tended to have a better ability than PP to predict Th-NCO. This result corroborates Bachman and coworkers’ study, which showed in an experimental model of VA-ECLS that the variation of CO_2_ elimination from the lungs correlated strongly with the variation of pulmonary blood flow [30]. Moreover, in our study, PaCO_2_ was measured as a control of effective CO_2_ removal by both lungs and ECLS. Owing to the facts that PaCO_2_ was in normal range and that sweep gas-to-blood flow ratio at ECLS as well as ventilator settings were unchanged, the EtCO_2_ changes followed the NCO quite well at low NCO levels. Indeed, in physiological conditions, when CO_2_ production is stable, the venous return allows CO_2_ transport to the lung where CO_2_ is removed from the blood through the regulation of pulmonary gas exchanges. EtCO_2_ is closely related to PaCO_2_, and the PaCO_2_-EtCO_2_ gradient is low (3–5 mmHg) [31]. An EtCO_2_ decrease, with parallel increase of PaCO_2_-EtCO_2_ gradient, while CO_2_ production or gas exchanges are unchanged, reflects quite directly a decrease in venous return and pulmonary artery flow, generating alveolar dead space [17, 18, 31, 32]. In the condition of VA-ECLS, a large amount of venous return is diverted to the membrane oxygenator by the ECLS circuit, but NCO keeps a fraction of the venous return for flow into the pulmonary artery towards the alveolar capillaries. Therefore, CO_2_ removal depends on ventilator and ECLS settings, but even with very low volume ventilation, EtCO_2_ variation parallels the changes in alveolar dead space related to low pulmonary artery blood flow. In our series, minute ventilation was low (median 3.6 L/min, which is roughly half the physiological value), but median PaCO_2_-EtCO_2_ gradient was 30 mmHg, i.e., 6-fold the normal range, when NCO was < 1 L/min. The EtCO_2_ kept going up when NCO increased above 1 L/min but the relation between EtCO_2_ and NCO curved down significantly when NCO exceeded 2 L/min (Fig. 2c), demonstrating that EtCO_2_ became independent on the NCO level when pulmonary vascular recruitment was completed. The limitation of EtCO_2_ monitoring is related to the access to exhaled CO_2_ that, in order to be trustable, needs the patient to be intubated.

Taken together, the two parameters offer a real-time, complementary monitoring method and could guide medical interventions (fluid loading, inotrope, cardiac pacing, VA-ECLS outflow titration, or left ventricle venting) for NCO optimization in situations of threatening NCO.

Our study has several limitations. It is a single-center nature study with a small number of patients and a strict control of lung and ECLS ventilations, and thus PaCO_2_. The ventilator and ECLS settings were fixed according to guidelines, which are routinely used in most ECLS centers. A quarter of the measures were made with concomitant left VAD support that induced missing data for PP. However, the association of transient or durable VAD with VA-ECLS is frequent and source of low NCO events. NCO monitoring is crucial in these patients and PP is not reliable. Based on previous observations [19], we thought that VAD do not impede interpretation of EtCO2/NCO relationship, which is why we chose to include patients with VAD. PAC monitoring was considered as the gold standard for cardiac output measurement but its accuracy in cases of low cardiac output and in the setting of VA-ECLS is disputed [27]. Although echocardiography was used for NCO < 1 L/min (failure of PAC assessment), cross measurements for comparison of the 2 techniques above 1 L/min would have been interesting. Finally, cardiac thrombosis and stroke incidences were not documented in this study, but recommended actions to prevent them (including LV venting) were applied when appropriate. The study was based on a physiological approach, and the small number of patients included would not allow any significant clinical assumption.

## Conclusions

The study shows that PP and EtCO_2_ are interesting parameters to monitor residual native cardiac output during VA-ECLS, specifically for detecting threatening NCO. Thresholds of PP < 15 mmHg and EtCO_2_ < 14 mmHg predicted NCO < 1 L/min with good accuracy. Further studies are needed to show if their utilization may help to implement therapeutic strategies in order to prevent thromboembolic and respiratory complications associated with VA-ECLS, and to improve patients’ prognosis.

## Data Availability

The datasets used and/or analyzed during the current study are available from the corresponding author on reasonable request.

## Abbreviations

AUC: Area under the curve
CS: Cardiogenic shock
EtCO_2_: End-tidal carbon dioxide
ICU: Intensive care unit
LV: Left ventricular
NCO: Native cardiac output
PAC: Pulmonary artery catheter
PAP: Pulmonary artery pressure
PAWP: Pulmonary arterial wedge pressure
PP: Pulse pressure
ROC: Receiver operating characteristics
SEC: Spontaneous echo contrast
Th-NCO: Threatening NCO
VA-ECLS: Veno-arterial extracorporeal life support
VAD: Ventricular assist device
